# A finite element analysis of a low-profile femoral neck system of screws in sleeves in a vertical femoral neck fracture model

**DOI:** 10.1186/s12891-024-07550-7

**Published:** 2024-06-06

**Authors:** Jun Sun, Le Wu, Nan Fang, Wenze Qiao, Lifeng Liu

**Affiliations:** Department of Trauma Orthopaedics, Shanghai East Hospital, Tongji University School of Medicine, 150 Jimo Road, Shanghai, 200120 China

**Keywords:** Femoral neck system, Femoral neck fractures, Finite element analysis

## Abstract

**Background:**

Femoral neck system (FNS) has exhibited some drawbacks, such as non-fit of the plate with the lateral femoral cortex, postoperative pain, and the potential risk of subtrochanteric fractures. We have developed a low-profile FNS system that addresses some compatibility issues in FNS. In this study, we conducted finite element analysis on the 1-hole FNS (1 H-FNS), 2-holes FNS (2 H-FNS), and low-profile FNS (LP-FNS) and compared their biomechanical performance.

**Methods:**

After the mesh convergence analysis, we established three groups of 1 H-FNS, 2 H-FNS, and LP-FNS. The interfragmentary gap, sliding distance, shear stress, and compressive stress and the bone-implant interface compression stress, stiffness, and displacement were determined under the neutral, flexion, or extension conditions of the hip joint, respectively. The stress and displacement of the femur after the implant removal were also investigated.

**Results:**

(1) There were no obvious differences among the three FNS groups in terms of the IFM distance. However, the LP-FNS group showed less rotational angle compared with conventional FNS (neutral: 1 H-FNS, -61.64%; 2 H-FNS, -45.40%). Also, the maximum bone-implant interface compression stress was obviously decreased under the neutral, flexion, or extension conditions of the hip joint (1 H-FNS: -6.47%, -20.59%, or -4.49%; 2 H-FNS: -3.11%, 16.70%, or -7.03%; respectively). (2) After the implant removal, there was no notable difference in the maximum displacement between the three groups, but the maximum von Mises stress displayed a notable difference between LP-FNS and 1 H-FNS groups (-15.27%) except for the difference between LP-FNS and 2 H-FNS groups (-4.57%).

**Conclusions:**

The LP-FNS may not only provide the same biomechanical stabilities as the 1 H-FNS and 2 H-FNS, but also have more advantages in rotational resistance especially under the neutral condition of the hip joint, in the bone-implant interface compression stress, and after the implant removal. In addition, the 1 H-FNS and 2 H-FNS have similar biomechanical stabilities except for the maximum von Mises stress after the implant removal. The femur after the LP-FNS removal not only is subjected to relatively little stress but also minimizes stress concentration areas.

## Highlights

We developed a low-profile FNS of screws in sleeves and demonstrated that the LP-FNS might not only provide the same biomechanical stabilities as the 1 H-FNS and 2 H-FNS, but also have more advantages in rotational resistance especially under the neutral condition of the hip joint, in the bone-implant interface compression stress, and after the implant removal.

The 1 H-FNS and 2 H-FNS groups had no notable difference in biomechanical stabilities except for the maximum von Mises stress after the implant removal.

Our study, for the first time, examined the stress and displacement of the femur after the implant removal and indicated that the femur after the LP-FNS removal not only was subjected to relatively little stress but also minimized stress concentration areas compared with the femur after the 1 H-FNS or 2 H-FNS removal.

## Introduction

Femoral neck fractures occur mostly in the elderly, while vertical femoral neck fractures (vFNFs) affect young adults (< 65 years old) in about 3% of cases; however, the treatment of young adults is a challenge in trauma orthopedics [1, 2]. High-energy trauma causes these fractures which are typically Pauwels type III fractures [3, 4]. Significant shear and rotational stresses at the fracture end due to the almost vertical angle make fixation extremely difficult and prone to complications such as fracture end displacement, osteonecrosis, and femoral head necrosis [5]. To date, the use of dynamic hip screws (DHS) or three parallel cannulated compression screws in an inverted triangular configuration is a standard method for the treatment of vFNFs [6, 7]. However, complications of vFNFs, such as nonunion, malalignment, femoral head necrosis, and failure requiring reoperation, remain high [3, 8, 9]. Recent advances include the DePuy Synthes femoral neck system (1-hole FNS, 1 H-FNS; 2-holes FNS, 2 H-FNS), which combines compressive and anti-rotational properties, offering minimal surgical trauma, resistance to anti-rotation and shear stresses, and effective compression of the broken end. Biomechanical tests indicate that the FNS exhibits superior stability compared to the traditional three cannulated screw fixation and effectively mitigates the risks of the trabeculae collapse and rotational failure [1, 10, 11].

**Fig. 1:**
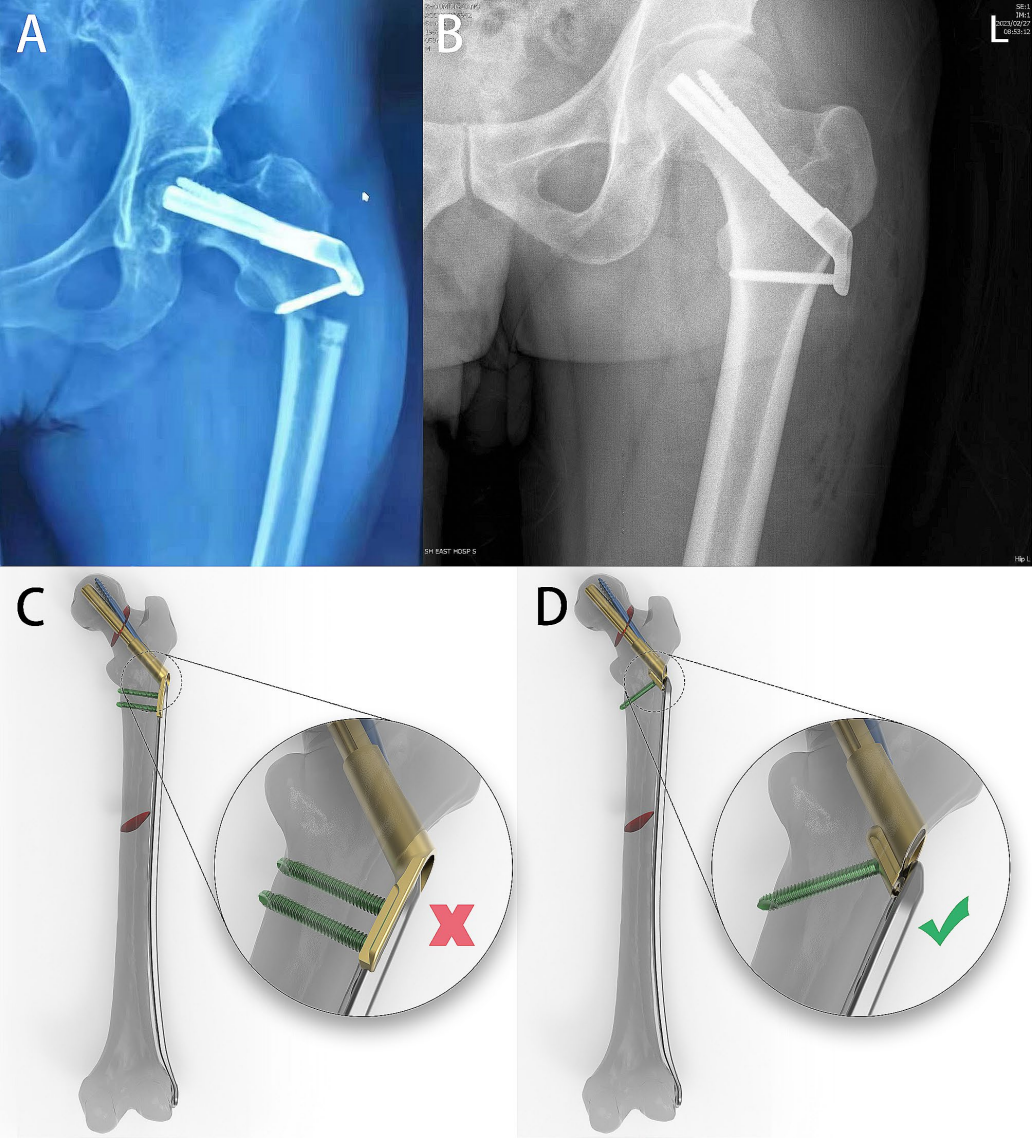
An image illustrating the drawbacks of FNS. (**A**) A patient who underwent FNS surgery experienced a re-fracture with locking of the nail hole after the operation. (**B**) X-ray images taken after FNS surgery revealed an obvious gap between the plate and femur. (**C**) FNS is not compatible with LISS for treating patients with combined fractures of the femoral neck and shaft. (**D**) LP-FNS can effectively be used in conjunction with LISS to treat patients with combined fractures of the femoral neck and shaft

Despite theoretical advantages and better biomechanical stabilities of the FNS, it also brought up some new issues in clinical practices, such as non-fit of the plate of the FNS with the lateral femoral cortex, postoperative pain, the potential risk of subtrochanteric fractures, and the incompatibility with femoral LISS (less invasive stabilization system) plate (The patient also has a combined femoral stem fracture) [12] (Fig. 1A and C). The ideal position of the FNS in the proximal femur is where the main nail of the FNS is located centrally or subcentrally to the femoral neck in the anteroposterior X-ray [1]. This design requires that both point and direction of entry of the guide wire on the proximal lateral femoral cortex must be accurate owing to the fixed angle of 130 degrees between the main bolt and the lateral plate of the sleeve of the FNS; otherwise, the tip of the guide wire must deviate from the center of the femoral head or the lateral plate of the sleeve must not fit with the lateral femoral cortex. Therefore, a hand-held guidance tool for the guide wire was designed to solve this problem, but in clinical practices it is not as simple as the theory suggests. The surgeon usually requires that the tip of the guide wire must be centrally or subcentrally located into the femoral head, but does not impose that the lateral plate of the sleeve must fit with the lateral femoral cortex. In fact, if the entry point and direction of the guide wire all are forced to be precise, it is likely that the position of the guide wire into the femoral neck will have to be adjusted several times, resulting in an increase in medical injuries and even fractures and a decrease in holding power of internal fixation. Additionally, the thick lateral plate (5 mm) can cause soft tissue irritation and subsequent aseptic inflammation and chronic pain in the long term [13]. A series of biomechanical studies [14, 15] indicate that subtrochanteric region is at risk for fractures, which is the entry point for the most distal locking screw of the FNS on the lateral femoral cortex. In summary, these new problems identified in clinical practices are mainly due to the lateral plate of the FNS.

Herein, we developed a low-profile FNS of screws in sleeves (LP-FNS) (Fig. 1D) and comparted it with conventional FNS using a finite element analysis (FEA) in a vFNF model. In this study, we will compare the interfragmentary motion (IFM), interfragmentary angle (IFA), and stress distribution of three groups of FNS in neutral, flexion, or extension states. This comparison aims to evaluate their biomechanical stability and rotational resistance. By examining these parameters, we intend to gain a deeper understanding of the performance and effectiveness of the FNS in various loading conditions. This study may provide a possible innovation for the FNS modification.

## Methods

### Construction of the finite element model

The study involved recruiting a volunteer, who had no history of hip and femoral fractures, metabolic bone disease, or any general comorbidities. The collection of femoral data has achieved approval from the Ethics Committee of Shanghai East Hospital. The volunteer has signed informed consent forms, demonstrating their understanding and agreement with the study procedures and potential risks involved. The experiment was conducted at our department, where we utilized the TOSHIBA Aquilion PRIME CT scanner (Canon Medical Systems Corporation, Tokyo, Japan) to scan the entire femur length with a 1 mm slice thickness, using a voltage of 120 KV, current of 125 mA, resolution of 512 × 512, and pixel spacing of 0.949/0.949. Following this, the CT data were exported into DICOM format and transferred to Mimics 21.0 software, where a mask was generated based on the CT images’ grayscale values. We utilized the “threshold” function in Mimics to automatically generate an initial mask. Two physicians manually cleaned and adjusted the mask using “edit mask” function. To accurately depict the femur as closely as possible. A preliminary 3D model of the femur was developed from the mask, and subsequently, in 3-Matic 13.0 software (Materialise, Leuven, Belgium), the model was removed nails by using local smoothing tool and trim tool. The model utilized a global smoothing tool to achieve an overall refinement. Using the STL format, the model was imported into Geomagic Wrap 2021 software (3D Systems, Rock Hill, USA) to repair any defects. To obtain the femoral geometry, a non-uniform rational B-splines (NURBS) surface was constructed, and surface fitting was performed.

### Surgical model validation and simulation

**Fig. 2:**
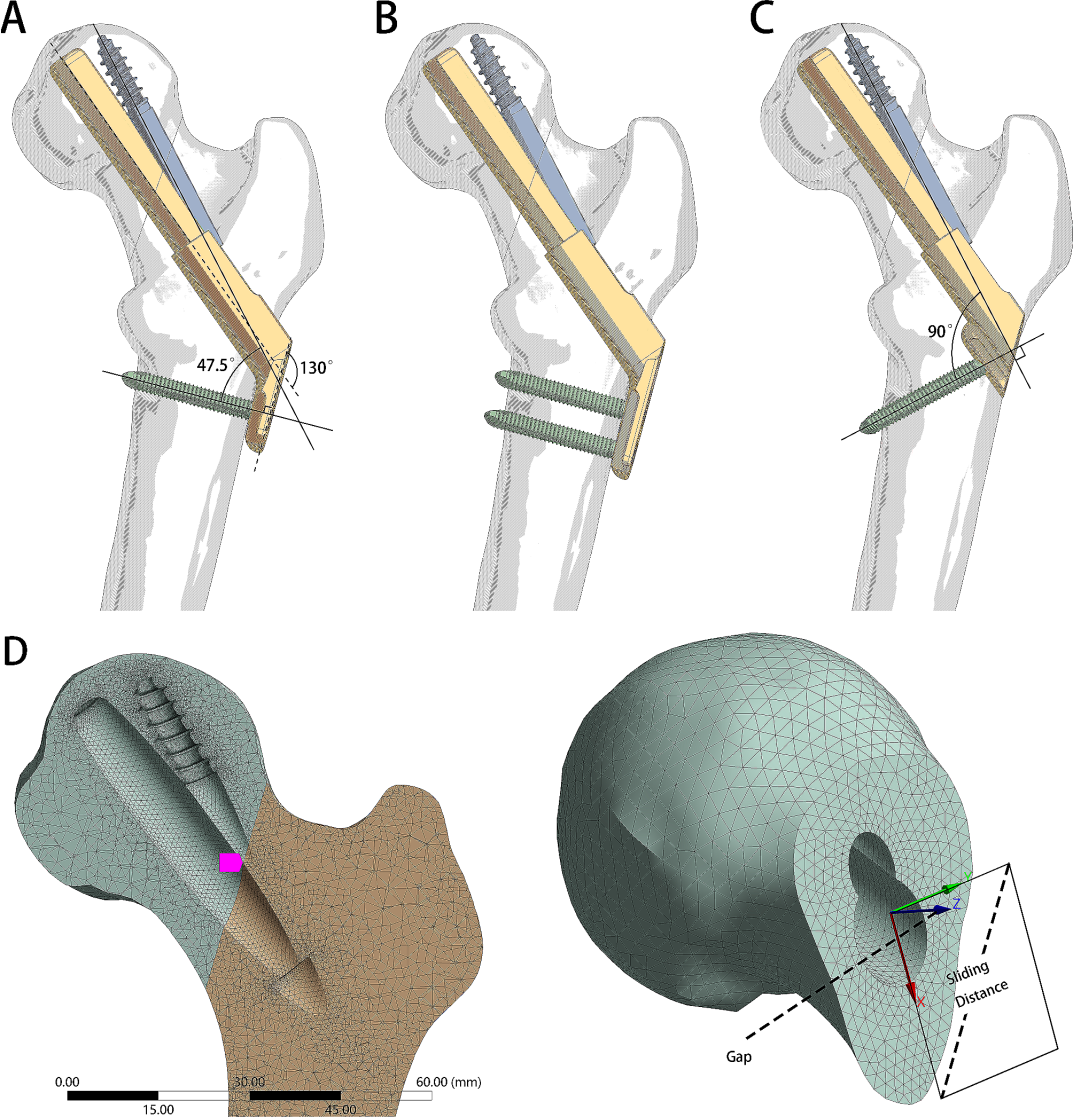
The preliminary diagrammatic drawings of the models. (**A-C**) The schematic diagrams of models (**A**) 1 H-FNS, (**B**) 2 H-FNS, and (**C**) LP-FNS. The angle between the anti-rotation screw and the locking screw is 47.5 degrees for both 1 H-FNS and 2 H-FNS, while it is 90 degrees for LP-FNS. (**D**) The schematic diagrams of the calculation method for Gap and Sliding Distance in the finite element model. Taking the centroid of the contact surface of the fracture as the origin, a coordinate system is established with an axis perpendicular to the fracture plane as Z-axis. The sliding distance of the fracture plane on XY-plane represents Sliding Distance, while separation along Z-axis represents Gap

The LP-FNS and FNS were designed and modeled in Spaceclaim 2022 R1 software (ANSYS Inc., Canonsburg, USA). The FNS model was created based on information from DePuy Synthes (DePuy-Synthes, West Chester, PA) and consisted of a plate with a 130° plate-to-bolt angle, which is available in 1-hole and 2-hole sizes, and a 10 mm diameter, 80 mm length bolt. The anti-rotation screw and locking screw had diameters of 6.4 mm and 5.0 mm, respectively. The LP-FNS sleeve was designed without the 5.0 mm thick plate, and a cylindrical projection was added to support the sleeve and secure the locking screw. All models were saved in step format for further use (Fig. 2A and C).

A femoral neck fracture model, classified as Pauwels III, was generated via the Spaceclaim software. The fracture plane was aligned at a 71° angle to the horizontal line of the coronal plane defined by the femoral neck axis and was orthogonal to the same coronal plane. Three internal fixation models, namely 1 H-FNS, 2 H-FNS, and LP-FNS, were developed based on clinical implant geometry data.

The processed femur and FNS were exported to step format and imported into Hypermesh 2022 software (Altair Engineering Inc., Troy, USA) for meshing. In this study, the 2D element type is set to trias, and the 3D element type is 4-node tetrahedral. The meshes were refined with proximity and curvature. The average element sizes for femur were set to 10 mm, 6.75 mm, 4.5 mm, 3 mm, 2 mm, 1.33 mm, and 0.8 mm, respectively. According to the experimental design, the meshes of different element sizes were exported to cdb format (ANSYS solver deck) and transferred to Mimics 21 (Materialise, Leuven, Belgium), where the HU value-based bone material properties were assigned to the meshes using empirical formulas. The material properties were assumed as isotropic, linear elastic, and inhomogeneous by assigning an individual Young’s modulus (E) to each element of the FE model using the CT scans. The empirical formula for assigning HU-based bone material properties is shown below [16, 17]:

1 $$\rho (g/c{m^3}) = 0.000968 \times HU + 0.5$$

2 $$\begin{array}{l}If\rho \le 1.2g/c{m^3},E\left( {MPa} \right) = 2014{\rho ^{2.5}},v = 0.2\\If\rho > 1.2g/c{m^3},E\left( {MPa} \right) = 1763{\rho ^{3.2}},v = 0.32\end{array}$$

The convergence of the mesh in the models was evaluated using ANSYS 2022 R1 FE simulation software (ANSYS Inc., Canonsburg, USA). Loading conditions were applied according to the hip joint force during normal walking, as reported by Bergmann et al. [18]. The distal femur model was constrained with zero degrees of freedom in all directions, and a force of 1400 N (about 250% of body weight) was applied to the femur surface in the direction from the femoral head center to the knee joint center to simulate the mechanical status during walking [19, 20]. The convergence behavior of FE bone models was measured using stiffness and stress and compared with previously published studies [10, 16, 21–23].

### Boundary conditions

Surgical models were subjected to a 1400 N force aligned with the femoral mechanical axis. In order to examine the anti-rotation capability of diverse implants, ± 10 Nm moments were imposed on the femoral head. The interface between the screw and plate and interface between the locking screw and femur was assigned a bonded contact, while the other interfaces were defined as sliding. The friction coefficient for the bolt to plate/sleeve was 0.2, while for bone-implant, it was 0.3, and for friction between fracture lines, it was set to 0.46 [16, 22, 24].

### Comparative parameters

**Fig. 3:**
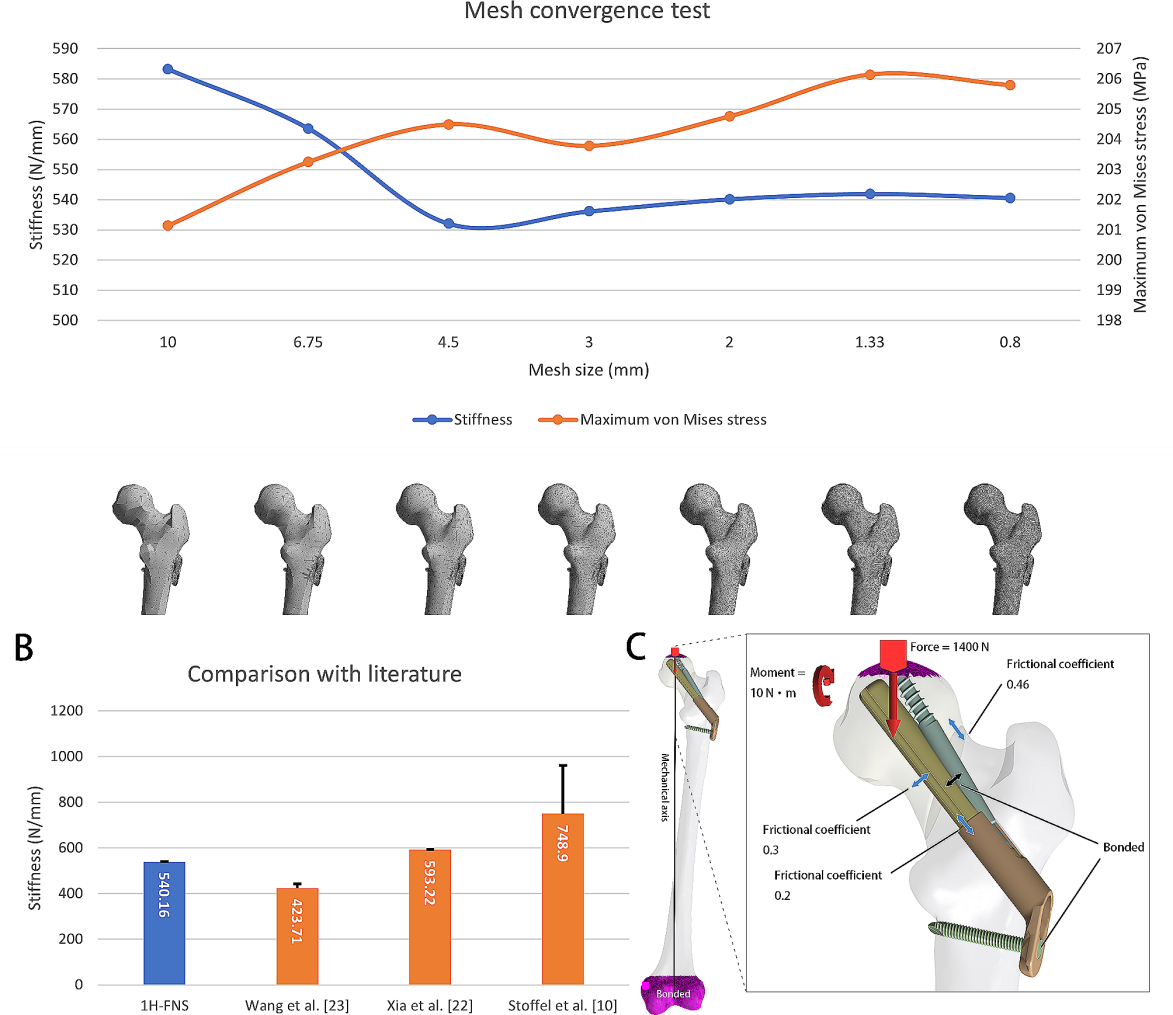
(**A**) Mesh convergence analysis of different FE mesh sizes. The findings of stress and stiffness exhibited gradual stabilization as the mesh size decreased, and a mesh size of less than 2 mm demonstrated superior accuracy. (**B**) The comparison between the results of 1 H-FNS and related experiments revealed that our study’s findings align closely with those of Xia et al. [22] The results fell within the range of Wang et al. [23] and Stoffel et al. [10], thus validating the suitability of our modeling method for further investigation. (**C**) Boundary conditions and loading force settings in the FNS group. Force: Loading force applied on the surface of the femoral head aligned with the femoral mechanical axis, which is equal to about 250% body weight. The interface between the screw and plate and interface between the locking screw and femur was assigned a bonded contact, while the other interfaces were defined as sliding. The friction coefficient for the bolt to plate/sleeve was 0.2, while for bone-implant, it was 0.3, and for friction between fracture lines, it was set to 0.46

Stiffness was calculated by dividing the patient-specific load by the displacement of the applied node (Fig. 3C). The determination of the relative displacement between fracture ends can be performed by utilizing the IFM-Calculator as a representation of the IFM [25]. In this context, the sliding distance denotes the amount of relative sliding displacement between the fracture ends, while the gap represents the extent of separation between the two ends (Fig. 2D). Based on the methodology provided by the IFM-Calculator, we established a local coordinate system. According to the definition of this local coordinate system, the Interfragmentary Angle (IFA) along the X-axis represents the anterior/posterior tilt of the femoral head, the IFA along the Y-axis represents the internal/external rotation of the femoral head, and the Z-axis represents the rotation of the femoral head.

## Results

### Model validation

During the mesh convergence analysis, we established the femur model integrated with the 1 H-FNS implant. The accuracy of the outcomes was assessed by implementing seven distinct FE mesh sizes. The findings of strain and stiffness exhibited gradual stabilization as the mesh size decreased, and a mesh size of less than 2 mm demonstrated superior accuracy (Fig. 3A). Therefore, in consideration of both the FE model size and result accuracy, a mesh size of 2 mm was considered sufficiently accurate. To validate the effectiveness of the finite element model, we compared the stiffness parameters with some previously published research, demonstrating that the model we have employed is justified (Fig. 3B; Table 1).

**Table 1:** Mesh convergence information

	Mesh size						
	10 mm	6.75 mm	4.5 mm	3 mm	2 mm	1.33 mm	0.8 mm
Element type	SOLID185	SOLID185	SOLID185	SOLID185	SOLID185	SOLID185	SOLID185
Nodes	273,951.00	278,236.00	285,492.00	311,384.00	334,898.00	662,838.00	2,028,880.00
Elements	1,365,097.00	1,389,238.00	1,426,362.00	1,569,165.00	1,668,065.00	3,588,261.00	11,583,041.00
Stiffness (N/mm)	583.226	563.589	532.066	536.153	540.165	541.922	540.569
Maximum von Mises stress (MPa)	201.145	203.249	204.496	203.784	204.760	206.143	205.790

### Comparison between LP-FNS, 1 H-FNS, and 2 H-FNS groups in values and distributions of interfragmentary gap, sliding distance, shear stress, and compressive stress

**Fig. 4:**
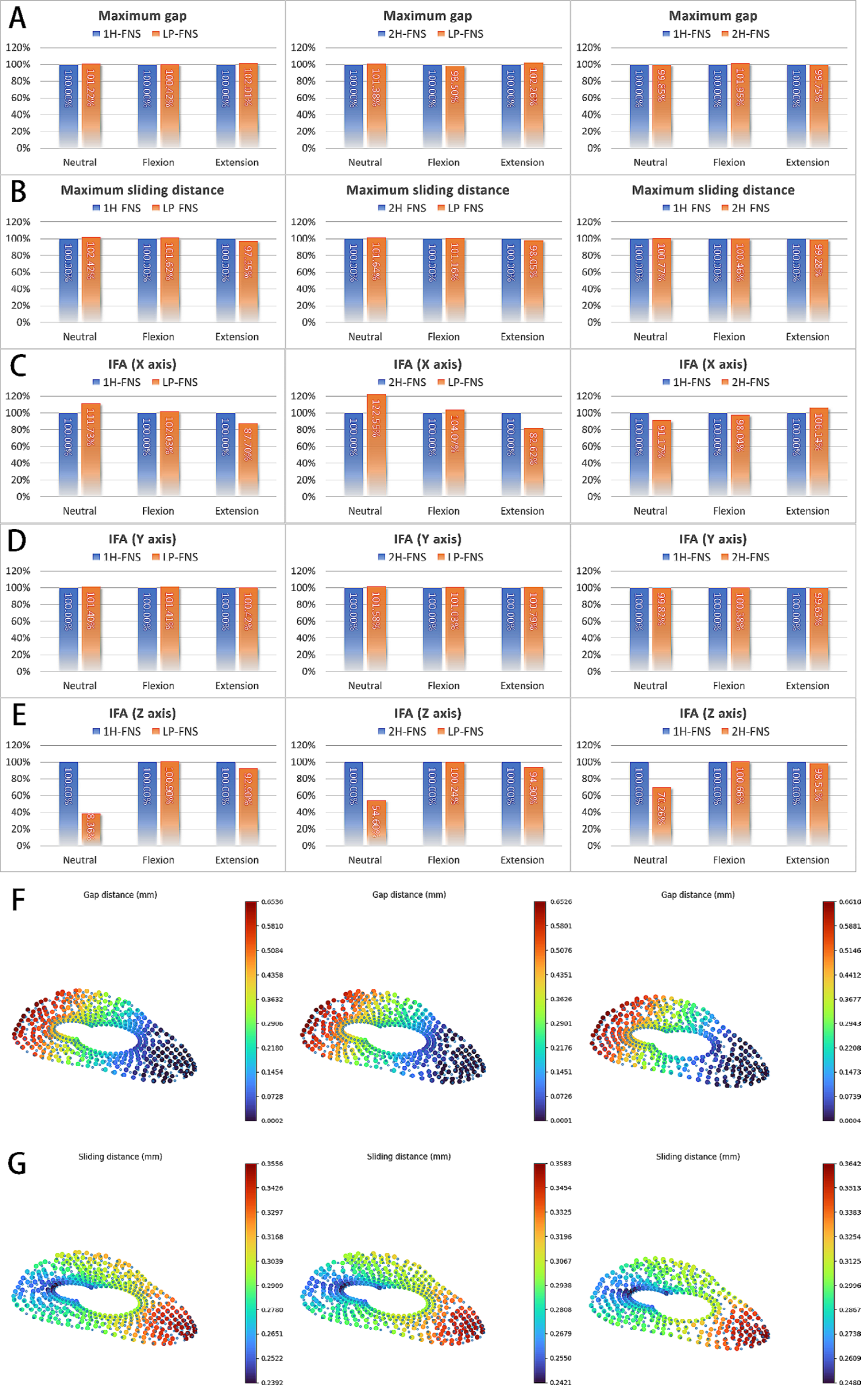
(**A-E**) Comparison charts of (**A**) maximum gap, (**B**) maximum sliding distance, (**C**) IFA in X axis, (**D**) IFA in Y axis and (**E**) IFA in Z axis between different groups. The Interfragmentary Angle (IFA) along the X-axis represents the anterior/posterior tilt of the femoral head, the IFA along the Y-axis represents the internal/external rotation of the femoral head, and the Z-axis represents the rotation of the femoral head. The left column represents 1 H-FNS vs. LP-FNS, the middle column represents 2 H-FNS vs. LP-FNS, and the right column represents 1 H-FNS vs. LP-FNS. (**F-G**) Distribution patterns of (**F**) gap and (**G**) sliding distance. The left column represents 1 H-FNS, the middle column represents 2 H-FNS, and the right column represents 2 H-FNS

In order to better interpret the IFM distance, it is divided into gap and sliding distance. In terms of the maximum gap value comparison, the 2 H-FNS group had the smallest gap, but the difference relative to the 1 H-FNS group was not obvious (1 H-FNS vs. 2 H-FNS: +0.15% in static, -1.92% in flexion, + 0.25% in extension). The LP-FNS group had the largest gap. (LP-FNS vs. 1 H-FNS: +1.22% in static, + 0.42% in flexion, + 2.01% in extension) (Fig. 4A and F). In terms of the maximum sliding distance comparison, the 1 H-FNS group demonstrated better performance, but the differences among the three groups were not obvious. (1 H-FNS vs. 2 H-FNS: -0.76% in static, -0.46% in flexion, + 0.72% in extension. LP-FNS vs. 1 H-FNS: +2.42% in static, + 1.62% in flexion, -2.65% in extension) (Fig. 4B and G).

**Fig. 5:**
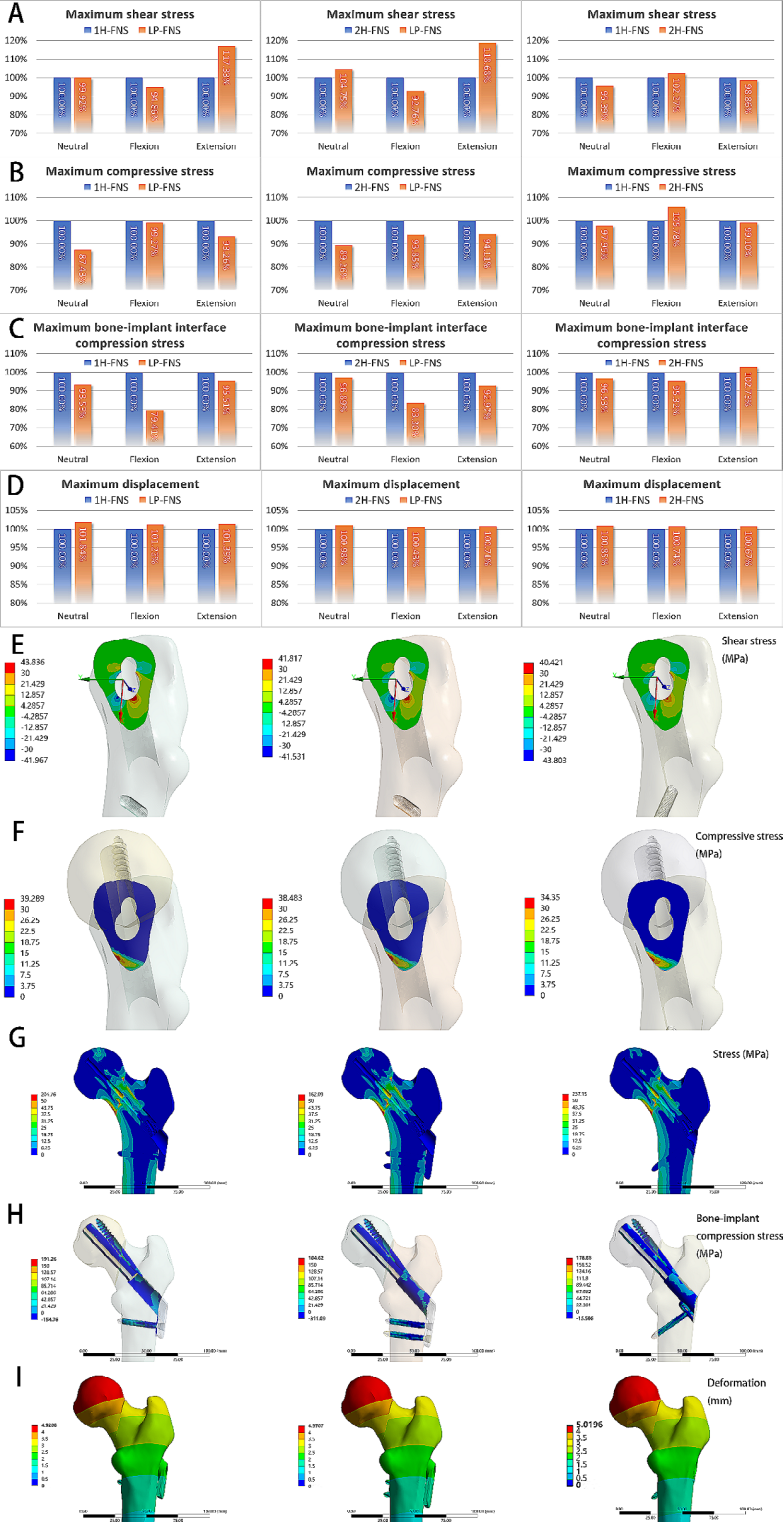
(**A-C**) Comparison charts of (**A**) maximum shear stress, (**B**) maximum compressive stress, (**C**) maximum bone-implant interface compression stress, and (**D**) maximum displacement between different groups. The left column represents 1 H-FNS vs. LP-FNS, the middle column represents 2 H-FNS vs. LP-FNS, and the right column represents 1 H-FNS vs. LP-FNS. (**E-I**) Distribution patterns of (**E**) shear stress, (**F**) compressive stress, (**G**) von Mises stress, (**H**) bone-implant interface compression stress and (**I**) displacement between different groups. The left column represents 1 H-FNS, the middle column represents 2 H-FNS, and the right column represents 2 H-FNS

Regarding the anterior/posterior tilt angle of the femoral head (X-axis IFA), the LP-FNS group performed poorly in the neutral position, with an angle increase of 11.73% and 22.55% compared to the 1 H-FNS and 2 H-FNS groups, respectively. However, it showed better stability in extension, with angle reductions of 12.30% and 17.38% compared to the 1 H-FNS and 2 H-FNS groups, respectively. There were no obvious differences among the three groups in flexion (Fig. 4C). There were no notable differences among the three groups in the internal/external rotation angle of the femoral head (Y-axis IFA, Fig. 4D). In terms of rotational resistance, the LP-FNS group demonstrated a clear advantage. In the neutral position, the rotation angle of the femoral head (Z-axis IFA) was reduced by 61.64% and 45.40% compared to the 1 H-FNS and 2 H-FNS groups, respectively. In extension, it was reduced by 7.10% and 5.70% compared to the 1 H-FNS and 2 H-FNS groups, respectively. There were no considerable differences among the three groups in flexion (Fig. 4E). The LP-FNS group demonstrated some advantages in terms of maximum compressive stress, with reductions of 12.57% and 10.74% compared to the 1 H-FNS and 2 H-FNS groups, respectively. However, there were no remarkable differences among the three groups in terms of maximum shear stress (Fig. 5A and B). These findings indicated that there are no obvious differences among the three FNS groups in terms of the IFM distance. However, the LP-FNS group demonstrated superior performance in rotational resistance and compressive stress.

The interfragmentary stress distribution patterns across the three models were observed to be consistent. Specifically, the interfragmentary gap was observed to be larger at the upper part of the fracture end and decreased towards the bottom, while the proximal end of the fracture exhibited slight sliding towards the bottom. Moreover, the maximum value appears in the inferior region of the fracture end and gradually decreases upward (Table 2; Fig. 5E and F).

**Table 2:** A summary of the FEA results

	Neutral			Flexion			Extension		
	1H-FNS	2H-FNS	LP-FNS	1H-FNS	2H-FNS	LP-FNS	1H-FNS	2H-FNS	LP-FNS
Nodes	334898	361480	302701	334898	361480	302701	334898	361480	302701
Elements	1668065	1828170	1521314	1668065	1828170	1521314	1668065	1828170	1521314
Averaged displacement (mm)	2.592	2.452	2.520	3.342	3.188	3.230	2.071	1.932	2.013
Maximum displacement (mm)	4.929	4.971	5.020	6.241	6.287	6.318	4.616	4.647	4.680
Stiffness (N/mm)	540.165	570.962	555.489	418.948	439.092	433.450	676.165	724.638	695.548
Averaged von Mises stress (Femur) (MPa)	6.668	7.263	5.979	7.101	7.686	6.477	7.055	7.619	6.328
Maximum von Mises stress (Femur) (MPa)	204.760	162.090	237.150	230.530	229.300	217.240	273.600	231.870	317.970
Maximum von Mises stress (FNS) (MPa)	146.830	156.160	66.402	150.100	119.680	104.020	105.970	153.950	97.045
Strain (Femur) (mm/mm)	0.029	0.025	0.035	0.029	0.031	0.029	0.039	0.031	0.045
Strain (FNS) (mm/mm)	0.048	0.042	0.021	0.050	0.035	0.028	0.048	0.040	0.029
Averaged bone-implant interface compression pressure (MPa)	1.609	1.609	1.270	1.798	1.858	1.545	1.725	1.727	1.410
Maximum bone-implant interface compression pressure (MPa)	191.260	184.620	178.880	217.590	207.410	172.780	233.600	239.980	223.120
Maximum gap (mm)	0.654	0.653	0.662	0.879	0.896	0.882	0.716	0.714	0.730
Maximum sliding distance (mm)	0.356	0.358	0.364	0.657	0.660	0.667	0.718	0.713	0.699
Maximum compressive stress (MPa)	39.289	38.483	34.350	75.320	79.671	74.773	35.940	35.616	33.518
Maximum shear stress (MPa)	43.836	41.817	43.803	71.891	73.523	68.197	68.981	68.196	80.935

### Comparison between LP-FNS, 1 H-FNS, and 2 H-FNS groups in values and distributions of bone-implant interface compression stress, stiffness, and displacement

There was a notable difference in the bone-implant interface compression stress under the neutral, flexion, or extension conditions of the hip joint between LP-FNS and 1 H-FNS or 2 H-FNS groups (compared with 1 H-FNS: average: -21.11%, -14.07% or -18.06%; maximum: -6.47%, -20.59%, or -4.49%; compared with 2 H-FNS: average: -21.08%, -16.83% or -18.17%; maximum: -3.11%, -16.70%, or -7.03%; respectively) (Fig. 5C); however, there were no obvious differences in the stiffness and maximum displacement (1 H-FNS: +2.84% and + 1.84%, + 3.46% and + 1.23%, + 2.87% and + 1.39%; 2 H-FNS: -2.71% and + 0.98%, -1.28% and + 0.48%, -4.01% and + 0.71%; respectively). In addition, when compared to the 1 H-FNS group, the 2 H-FNS group showed no considerable changes in maximum bone-implant interface pressure and displacement (neutral: -3.47% and + 0.85%; flexion: -4.68% and + 0.74%; extension: +2.73% and + 0.67%, respectively). However, the 2 H-FNS group exhibited increased stiffness (neutral: +5.70%; flexion: +4.81%; extension: +7.17%) (Fig. 5D).

The distribution patterns of bone-implant interface compression stress, stiffness, and displacement across the three models were observed to be consistent (Fig. 5H and I). The fracture line exhibited remarkable stress concentration at both ends, mainly occurring in the inferior region of the femoral neck and around the implant. Stress concentration was also evident at both ends of the locking screw canal. In other locations, stress uniformly decreased inward along the bone cortex. The FNS concentrated stress on the bolt and anti-rotation screw, particularly at the junction of the bolt and anti-rotation screw, near the shear stress concentration of the fracture line, and at the thread of the anti-rotation screw (Table 2; Fig. 5G).

### Comparison between LP-FNS, 1 H-FNS, and 2 h-FNS groups in values and distributions of stress and displacement of the femur after the implant removal

**Fig. 6:**
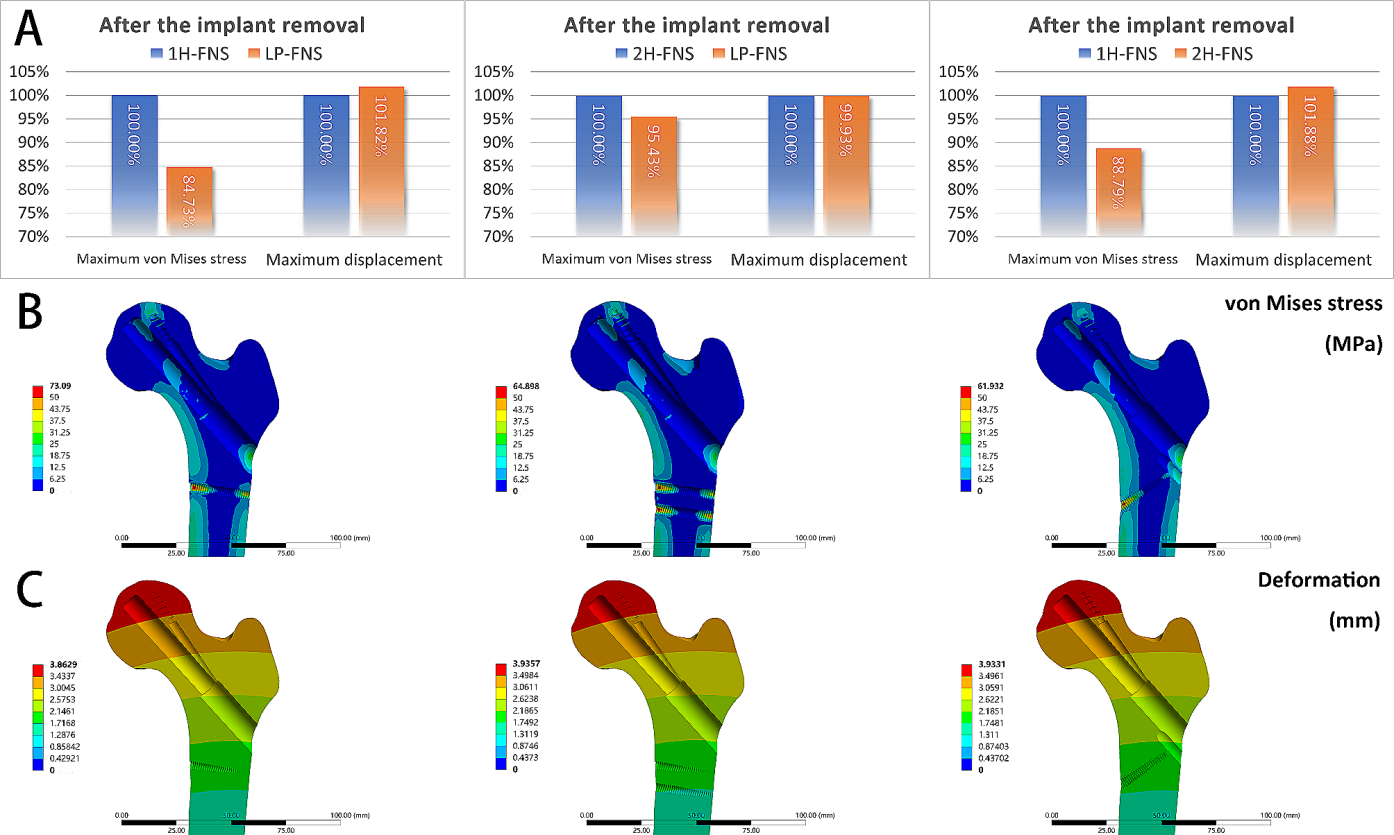
(**A**) Comparison charts of Maximum von Mises stress and Maximum displacement after the removal of the implant. The distribution patterns of (**B**) von Mises stress and (**C**) displacement after the removal of the implant is illustrated blow. The left column represents 1 H-FNS, the middle column represents 2 H-FNS, and the right column represents 2 H-FNS

Since the design of the LP-FNS sleeve increases the damaged areas of the proximal lateral femoral cortex, possibly resulting in localized stress concentrations that will increase the risk of fractures, we investigated values and distributions of stress and displacement of the femur after the implant removal. In order to simulate the situation after the implant removal, we removed the internal fixation and stabilized the fractured bone. After the implant removal, there was no meaningful difference in the maximum displacement between LP-FNS, 1 H-FNS, and 2 H-FNS groups (LP-FNS vs. 1 H-FNS: +1.82%; LP-FNS vs. 2 H-FNS: -0.07%; 2 H-FNS vs. 1 H-FNS: +1.88%), but the maximum von Mises stress displayed a notable difference between 1 H-FNS and LP-FNS or 2 H-FNS groups (LP-FNS vs. 1 H-FNS: -15.27%; 2 H-FNS vs. 1 H-FNS: -11.21%) except for the difference between LP-FNS and 2 H-FNS groups (-4.57%) (Fig. 6A). These findings suggest that the femur after the LP-FNS removal is subjected to relatively little stress.

After the implant removal, stress distribution in the three models was quite similar, with stress mainly concentrated at both ends of the locking screw canal. As the LP-FNS set the entrance of the locking screw at the inferior part of the sleeve, an additional opening was formed on the lateral cortex of the femur. Interestingly, this design minimized stress concentration areas for the LP-FNS group after the internal fixation removal as compared to 1 H-FNS and 2 H-FNS groups. In addition, stresses near the entrance of the FNS remained unchanged, whereas stresses near the fracture site noticeably decreased after the internal fixation removal. However, stresses at both ends of the locking screw canal remarkably increased (Fig. 6B). The deformations of the three model groups did not exhibit obvious distinctions (Fig. 6C).

## Discussion

Despite the favorable biomechanical properties of the FNS in treating femoral neck fractures, its rear plate may not fit snugly into the lateral femoral cortex of all individuals [26], resulting in notable gaps and potential biomechanical issues associated with the external screw-plate design [11]. Stassen et al. reported that about 23.5% of patients experienced persistent hip pain within a year, possibly due to the external plate [13]. Moreover, two of these patients had no radiological abnormalities after fracture healing at postoperative eight months but complained of the sensation of irritating osteosynthesis material [13]. Therefore, we have modified the plate of the FNS based on the concept of a low-profile FNS of screws in sleeves.

### Comparison between LP-FNS and 1 H-FNS or 2 H-FNS groups

We compared the differences between LP-FNS and 1 H-FNS or 2 H-FNS groups using the FEA and found no obvious differences in the maximum interfragmentary gap and sliding distance, in the stiffness and maximum displacement, and in the maximum interfragmentary shear stress under the neutral or flexion conditions except for the extension, which may be attributed to the use of the same sliding compression system. Cha et al. [27] conducted measurements on the gap and sliding distance of 2 H-FNS. In their study, they obtained smaller values for both the gap and sliding distance compared to the findings in our current research (maximum gap: 0.19 mm vs. 0.65 mm; sliding distance: 0.15 mm vs. 0.70 mm). Cha et al. utilized a higher bone-implant friction coefficient (0.42) and employed different measurement techniques, which we believe to be the underlying reasons for the disparity observed in the gap and sliding distance. Since people rarely hyperextend their hips in real life, this indicator (the extension), even though obvious different, has very limited impact on the conclusions. It is noteworthy that the LP-FNS exhibited superior rotational stability during the IFA testing, which we attribute to the larger angle between the anti-rotation screw and the locking screw. In addition, according to the stress distribution maps, the interfragmentary compressive stresses in all three groups were concentrated on the lower part of the fracture surface. This compressive stress formation is mainly due to the downward angulation caused by the strain of the femoral head under body weight, while the proximal fracture end compresses the distal fracture end during the sliding compression process, resulting in prominent stress concentration.

However, the maximum interfragmentary compressive stress and the bone-implant interface compression stress were decreased in LP-FNS group compared with 1 H-FNS or 2 H-FNS groups. The strength of fracture fixation is associated with the strength of the bone. Patients with osteoporosis or poor cortical bone are more prone to excessive compression and absorption of the fracture ends at the early stage during fracture healing, leading to the femoral neck shortening [28]. The postoperative femoral neck shortening is mainly related to anatomical characteristics and mechanical environment [29, 30]. Therefore, the remarkable stress concentration at the fracture ends is detrimental to the recovery of femoral neck fractures. If the stress concentration is too great, it can lead to collapse of the trabeculae in the femoral neck, and the long-term stress concentration may result in the femoral neck shortening. Although the FNS can effectively reduce femoral neck shortening compared with cannulated cancellous screws [1, 11, 31], the LP-FNS may be more effective in reducing the occurrence of this complication. In addition, the lower bone-implant interface compression stress in the LP-FNS indicates smoother stress transfer and reduces the likelihood of loosening of the internal fixation after long-term implantation.

Besides, in vitro studies have shown that only 25% of the stress in femoral neck fractures is borne by the internal fixation device, while 75% of the stress is borne by the femur itself [21]. So, even without weight-bearing on crutches, the fracture ends still experiences greater pressure due to the muscles maintaining body balance, increasing the risk of nonunion. In this study, the LP-FNS exhibited lower average femoral stress compared to the 1 H-FNS or 2 H-FNS in the neutral, flexion, and extension states. A smaller average femoral stress implies less stress on the trabeculae, making microfractures less likely to occur, which may have a more positive impact on reducing the risk of nonunion. Although the maximum femoral stress in the LP-FNS showed an increase in some cases, these maximum stresses were concentrated at mesh mutations or interfaces and the maximum stress values only existed in small areas. As for the reason, we believe these areas are stress singularities or the compression between fracture surfaces. Additionally, a concentration of stress implies a greater risk of fractures. It is worth noting that our study’s stress distribution is similar to many studies, the stresses of internal fixation are remarkably higher than the stress of the femur, which differs from our research. Upon comparison, we have discovered that the contact between the threads of their anti-rotation screw and the femur is considered a bonded contact, resulting in the direct transmission of forces to the internal fixation. This is evident from the stress distribution observed in related studies, where the stress on the threads of the anti-rotation screw is not pronounced [22, 31–33]. Moon et al. found the FNS could cause the refracture near the locking screw hole in biomechanical test [15], and we have also encountered such cases in clinical practice, indicating the likely presence of stress concentration around the locking screws. This also is confirmed by our results of the stress distribution patterns. All three groups showed stress concentrations at both ends of the locking screws, but it is worth noting that the LP-FNS, due to the design of screws in sleeves, only had one stress concentration point, and its stress was also lower, which is helpful for reducing the risk of the refracture.

### Comparison between 1 H-FNS and 2 H-FNS groups

A biomechanical study conducted by Fan et al. [34] indicate that both the 1 H-FNS and 2 H-FNS are equally effective in treating femoral neck fractures with Pauwels angles less than 60°, but in cases of vFNFs at angles greater than 70°, the use of the 2 H-FNS is more effective. The trends of their results are consistent with our findings that the 2 H-FNS is more effective in initial stability than the 1 H-FNS (The difference is greater than 5%, but less than 10%), but there was no obvious difference in the stability of bone fragments. Although the 2 H-FNS provides improved initial stability, it is not without its drawbacks. For instance, the use of longer plates and larger incisions can lead to greater complexity in placement and an extended operative duration. It is worth noting that a stress concentration was observed at the locking screw located on the lateral cortex of the femur, especially in the lower locking screw of the 2 H-FNS. We believe that the additional locking screw design causes disruption in the continuity of the femoral cortex, and a longer lever arm leads to stress concentration.

### Comparison between LP-FNS, 1 H-FNS, and 2 h-FNS groups after the implant removal

Our previous investigation demonstrated a plausible association between femoral load caused by incorrect screw removal and the incidence of osteonecrosis of the femoral head (ONFH) or femoral neck refracture [35]. Furthermore, the design of the LP-FNS causes more damage to the lateral cortex of the femur due to the locking screw in the sleeve. So, we investigated values and distributions of stress and displacement of the femur after the implant removal. We found that there was no difference in the maximum displacement and stress distribution between LP-FNS, 1 H-FNS, and 2 H-FNS groups, but the maximum von Mises stress displayed a meaningful difference between them, suggesting that the femur after the LP-FNS removal is subjected to relatively little stress. Moreover, the design of the LP-FNS may minimize stress concentration areas after the internal fixation removal. Hence, after the implant removal, the femur of the LP-FNS group has better biomechanical advantages and is less likely to be refractured compared to the 1 H-FNS or 2 H-FNS groups.

### Limitations

This article still has some limitations. We did not investigate the influence of first-order and second-order elements on the finite element analysis. The femur loading only considered the initial stability; other conditions corresponding to daily activities and the gait cycle are not evaluated. Additionally, the influences of surrounding muscles and ligaments on femur are ignored, although the effect of muscles is very small when standing position is chosen [36]. A stress singularity was observed at the junction of the femur and the implant, where stress values become unbounded [37]. There is an abrupt shape change at this location, which a locally dense mesh can solve. Nevertheless, we verified the convergence of the mesh, and the results were explored in various ways to ensure the study’s validity. In all, our study is based on a FEA without clinical evidence, which could just be a hypothesis and cannot be definite evidence.

## Conclusion

In summary, we developed a low-profile FNS of screws in sleeves and based on a FEA, for the first time, demonstrated that the LP-FNS might not only provide the same biomechanical stabilities as the 1 H-FNS and 2 H-FNS, but also have more advantages in rotational resistance especially under the neutral condition of the hip joint, in the bone-implant interface compression stress, and after the implant removal. Also, our study examined the stress and displacement of the femur after the implant removal and indicated that the femur after the LP-FNS removal not only was subjected to relatively little stress but also minimized stress concentration areas. In addition, the 1 H-FNS and 2 H-FNS groups had no obvious difference in biomechanical stabilities except for the maximum von Mises stress after the implant removal.

## Data Availability

The datasets generated during and analyzed during the current study are not publicly available because some data of the project are not suitable for disclosure but are available from the corresponding author on reasonable request.
